# The intensive outreach programme for child and adolescent eating disorders: an uncontrolled case series of clinical characteristics and treatment outcomes

**DOI:** 10.1186/s40337-026-01664-0

**Published:** 2026-06-29

**Authors:** Cliona Brennan, Ellen McAdams, Elena Pears, Paula Herrera-Gener, Julian Baudinet, Mima Simic

**Affiliations:** 1 Maudsely Centre for Child and Adolescent Eating Disorders, South London and Maudsley NHS Trust, Denmark Hill, London, SE5 8AZ UK; 2 Department of Nutritional Sciences, Franklin-Wilkins Building, Kings College London, London, UK; 3 Department of Nutrition and Dietetics, London Metropolitan University, Holloway Road, London, UK; 4 Centre for Research in Eating and Weight Disorders (CREW), Institute of Psychiatry, Psychology and Neuroscience, King’s College London, De Crespigny Park, London, SE5 8AZ UK

**Keywords:** Child, Adolescent, Eating disorders, Anorexia nervosa, Intensive outpatient programme

## Abstract

**Introduction:**

Despite growing interest, empirical evidence remains limited for the efficacy of intensive outpatient programmes (IOPs) for eating disorders (EDs), particularly outside the USA. No prior studies have examined the delivery or effectiveness of IOPs for children and young people in the UK. This study addresses this gap by presenting pilot data from a UK-based community IOP embedded within an existing child and adolescent ED service.

**Methods:**

We conducted a prospective cohort study of young people aged 12–18 years referred to the IOP at the Maudsley Centre for Child and Adolescent Eating Disorders between February 2024 and August 2025. Eligible participants had a diagnosis of an ED according to ICD-11 criteria, or disordered eating requiring acute admission, and completed the IOP treatment. Data were collected on referral pathways, demographic and clinical characteristics, treatment course, and outcomes, including change in weight and self-reported ED/mental health symptoms.

**Results:**

During the recruitment period the IOP served a heterogeneous cohort of 68 young people with high rates of psychiatric and neurodevelopmental comorbidity. Treatment delivery included outreach on paediatric wards, intensified outpatient treatment, and consultation with wider care networks. Mean duration of the IOP was 24 days (SD = 10.7). Most participants demonstrated improved weight trajectory (mean weight gain = 4.5% mBMI, SD = 6.8, *p* < 0.001). ED symptom psychopathology did not significantly improve over the short IOP duration.

**Conclusion:**

Findings indicate that a brief IOP intervention is effective for weight restoration in underweight adolescents with EDs, functioning as both an intensification of outpatient treatment and an effective way of keeping young people in the community who are at high risk of an inpatient admission. Future research would benefit from controlled designs, explore long-term outcomes, and examine adaptations for neurodiverse populations.

**Supplementary Information:**

The online version contains supplementary material available at 10.1186/s40337-026-01664-0.

## Abstract (plain language summary)

Eating disorders in children and young people are serious mental health conditions that often require intensive treatment. Intensive outpatient programmes (IOPs) provide structured, frequent treatment while allowing young people to remain at home. Although IOPs are increasingly used, there is little research on how effective they are, particularly outside the United States. No previous studies have examined IOPs for young people with eating disorders in the United Kingdom. This study reports early findings from a pilot IOP delivered within a specialist child and adolescent eating disorder service in the UK.

We followed a group of young people aged 12–18 who were referred to the programme between February 2024 and August 2025. Participants had a diagnosed eating disorder, or severe disordered eating that might otherwise require hospital admission. We collected information on referral routes, patient characteristics, how the treatment was delivered, and changes in weight and self-reported eating disorder and mental health symptoms.

Sixty-eight young people took part. Many had additional mental health or neurodevelopmental conditions. On average, treatment lasted about 24 days. Most participants showed improved weight gain during the programme, but eating disorder thoughts and feelings did not improve significantly over this short period.

Overall, the programme helped support weight restoration and may help prevent hospital admission by providing more intensive treatment in the community. Further research should examine long-term outcomes and how programmes can better support neurodiverse young people.

**Supplementary Information:**

The online version contains supplementary material available at 10.1186/s40337-026-01664-0.

## Introduction

Feeding and eating disorders (EDs) are severe psychiatric conditions with multifactorial biopsychosocial aetiologies [20, 59]. They carry some of the highest rates of morbidity and mortality among psychiatric illnesses, underscoring their clinical seriousness [42, 49]. Adolescent ED treatment pathways emphasise timely assessment, early family engagement, and systematic monitoring of weight gain as a key prognostic indicator [31, 43, 51]. A shorter duration of untreated illness and rapid initiation of evidence-based interventions are consistently associated with improved outcomes [31, 48, 55].

When medically safe, outpatient treatment is preferred for adolescents as they are able to remain at home and with their support networks, with positive effects broadly cited in the literature. For cases where remission is not achieved in standard outpatient treatment, more intensive treatment options, such as inpatient care [41], day programmes (DPs) [9], Partial Hospitalisation Programmes (PHPs) [57], intensive multi-family therapy [4, 5] and intensive outpatient programmes (IOPs) [1], are often used to facilitate change.

**Table 1:** Comparison of IOP, DP/PHP, residential and inpatient settings for ED

	IOP	DP/PHP	Residential	Inpatient
Professionals	Multi-disciplinary	Multi-disciplinary	Multi-disciplinary	Multi-disciplinary
Interventions	Psychosocial, nutritional, medical	Psychosocial, nutritional, medical	Psychosocial, nutritional, medical	Psychosocial, nutritional, medical
Modality	Individual care	Individual and group-based care	Individual and group-based care	Individual and group-based care
Setting	Community and paediatric wards	Community	Residential unit	Inpatient ward
Duration	~ 2–6 weeks	~ 3–18 weeks	~ 11–12 weeks	> 12 weeks
Frequency	4 to 7 days per week	4 to 7 days per week	24 h/day, 7 days per week	24 h/day, 7 days per week

Inpatient admission is typically reserved for cases of acute medical instability, elevated psychiatric risk, or lack of treatment response [52]. In the UK, inpatient treatment for young people with eating disorders (EDs) typically follows two main pathways: short-term paediatric admissions, lasting days to weeks, to manage acute medical instability, and longer-term admissions to specialist ED units (SEDUs) for intensive psychiatric care, often lasting several months [32]. While SEDUs provide structured multidisciplinary input to support weight restoration [36], interrupt maladaptive eating cycles [22], and address risks such as self-harm and suicidality [25], they are costly, disruptive to family life, and frequently associated with poorer outcomes than community-based alternatives [13, 28, 47].

DPs have equivalent outcomes to inpatient treatment [39] and may have better outcomes at follow-up [38]. Additionally, they are more cost effective than inpatient admissions for young people with EDs [30]. As such, they are becoming increasingly widespread internationally. Like DPs, PHPs are shown to have positive effects on clinical outcomes [33, 35, 50]. PHPs that specifically integrate evidence-based family therapy models, are particularly effective in improving outcomes, supporting improved weight trajectory and reduction in number and length of admissions [35]. MFT for EDs, a group and family-based intervention, was designed to optimize treatment outcomes by mitigating perceptions of isolation and stigma, strengthening familial relationships, and facilitating the development of family skills [4, 29]. Some models of MFT for ED aim specifically to intensify treatment in the initial phases to promote improved outcomes in young people with ED [11].

While DPs/PHPs have been shown to be an effective way of intensifying outpatient treatment and reducing inpatient admissions, they are resource intensive, last several weeks to months and may have waiting lists. Intensive outpatient programmes (IOPs) offer a more flexible, briefer intervention that sits between routine outpatient care and DPs/PHPs [1], for comparison table for DP vs. IOP see Table 1 and previous publication [17]. IOPs provide rapid, structured intervention during periods of acute crisis, aiming to prevent escalation to higher levels of care, while enabling young people to remain engaged in home and educational environments [41]. IOPs may have a key role in in reducing the frequency and duration of paediatric hospital admission and/or SEDU admission in adolescent EDs [24] and likely improve weight trajectory for adolescents who are not advancing in out-patient treatment, although research investigating the impact of IOP on treatment outcomes is greatly lacking. Compared with repeated paediatric admissions, which increase the likelihood of subsequenttransfer to SEDUs [32], IOPs can offer earlier, outpatient-based intensification of care in an individualised way. Core components typically include frequent therapeutic sessions, meal support, and close medical monitoring. By combining responsiveness with a shorter duration than DPs, IOPs may support weight restoration, reinforce recovery within the family, and consolidate treatment gains in everyday contexts [11, 15].

Although international interest in IOPs is growing [24, 41], evidence remains sparse. In the UK, there are currently no published empirical studies examining the delivery or outcomes of IOPs for young people with EDs. Available data on the young person, parent and clinican experience of IOPs is promising and suggests they are perceived as supportive and a useful way to intensify treatment [16, 17]. We aim to address this gap by presenting quantitative data from a pilot IOP embedded within a UK community ED service for children and young people.

We had four primary objectives:

1. To describe the IOP pathway, including frequency of referral from each source and onwards journey of care.
2. To characterise the demographic and clinical profile of young people referred to the IOP, including diagnostic breakdowns (and main comorbidities);
3. To describe the treatment course, including duration, intensity, and rate of subsequent hospital admission; and.
4. To evaluate treatment impact, specifically changes in weight (and self-reported ED symptoms/ mental health difficulties) from IOP assessment to discharge. Weight change was also reported for the five weeks following discharge from IOP.

## Methods

### Ethical considerations

This project was granted ethical approval by the South London and Maudsley Child and Adolescent Mental Health Services (CAMHS) service evaluation and audit committee (approval number 330, 23 August 2023).

### Treatment description

This was a prospective cohort study conducted at the Maudsley Centre for Child and Adolescent Eating Disorders (MCCAED) at the Maudsley Hospital in London, United Kingdom. The IOP at MCCAED is for children and adolescents under 18 years of age, presenting with ED symptoms. It was developed within the specialist outpatient team at MCCAED in 2024. The IOP is not a standalone treatment, but is embedded within a comprehensive outpatient ED service that has expertise in ED focused family therapy [11, 31] and other evidence-based treatments for ED such as Radically Open Dialectical Behaviour Therapy [12]. The IOP intervention is individualised to each family and based off the collaborative formulation developed [10]. Sessions are provided by a multidisciplinary team that is equipped to manage severe physical and psychiatric risk. No IOP team member works solely in IOP, with all team members having a case loads/ duties in other parts of the wider MCCAED service. This supports integration within the wider service, as well as flexibility to increase or decrease IOP time depending on the clinical need. 

Young people (YP) are referred to the IOP when there is a deterioration in physical health parameters set out by UK ED national guidance [53]. Two routes of referral to the IOP exist for these YP;

1. Via the MCCAED service whereby the young person has already been assessed and diagnosed with an ED and despite actively receiving treatment at MCCAED, continues to deteriorate.
2. Triggered by admission to a local paediatric ward (via the respective emergency department) due to medical instability arising as a result of a suspected (but undiagnosed) ED.

The latter route of referral necessitating a comprehensive ED assessment to confirm or deny the suspected ED diagnosis. In this sub-set of YP admitted on the paediatric ward, the IOP provides brief formulation driven interventions prior to the ED diagnosis being confirmed, with the aim of expediting discharge back to community care (where the comprehensive ED assessment takes place if still indicated).

IOP aims to help the YP and their family to arrest deterioration in health, reducing length of hospital stay (for those already admitted to the paediatric ward) and supporting ongoing community treatment, as opposed to moving onwards to SEDU inpatient care.

Prior to commencing the IOP most young people express low motivation to recover, have multiple maintaining factors for restrictive ED and their parents may have been struggling to provide consistent support at home. Dietary restriction leading to ongoing weight loss and physical symptoms of malnutrition are common in most YP.

The IOP team is comprised of specialist mental health nurses, a consultant child and adolescent psychiatrist, clinical psychologist, an assistant psychologist, and a dietician. The duration of the program is typically four to five weeks, Monday through Friday, with flexibility to shorten or lengthen treatment where clinically needed (i.e., some YP may complete their IOP treatment within a shorter duration of 1 to 3 weeks or extend their IOP treatment beyond 4 weeks where needed)The IOP intensity of attendance is decided on a case-by case basis according to clinical need. Most young people begin the IOP on a full-time basis with a clear plan of reducing days in the IOP each consecutive week.

The IOP treatment begins with daily face-to-face sessions in the first week and gradually transitions to less frequent contact over subsequent weeks, including virtual or telephone check-ins by the final week. The team adopts a family-centered approach to treatment, with a primary focus on physical health restoration (e.g., weight stabilisation or restoration) and introduction of regular eating. This is tailored to meet the individual needs of each young person, often intensifying the support already being provided by the outpatient ED service. Common themes for the IOP sessions include practical meal support and planning, parent coaching, goal setting and motivational work, distress tolerance skills, and enhanced physical monitoring; all with the overarching aim of preventing or reducing the length of hospital admission. A joint session takes place between the family, IOP clinician and MCCAED outpatient/care-coordinating therapist at least once each week to establish and review shared goals for IOP, review treatment progress and collaboratively plan ongoing treatment. Please see supplementary information for more specific details on weekly activities that take place in the IOP.

### Sample

Young people (age 12–18 years) who were referred to the IOP and had (1) a diagnosis of an eating disorder according to the International Classification of Diseases, 11th Revision (ICD-11) (World Health Organization, 2019), namely anorexia nervosa, bulimia nervosa, avoidant restrictive food intake disorder, or otherwise specified feeding or eating disorder or, in a smaller number of cases, presented with disordered eating symptoms in the context of another mental health disorder only when these symptoms had led to an admission to an acute paediatric ward, and (2) completed the IOP treatment between February 2024 and August 2025 were eligible for this study.

Young people who were not underweight, and were diagnosed with bulimia nervosa or otherwise specified feeding or eating disorder, that were included required IOP support to regulate eating (due to restricted, dysregulated or disordered eating patterns leading to weight loss, low weight and/or physical instability), and to stabilise or gain weight (due to being on a weight loss trajectory, resulting in impaired physical health parameters). Patients with binge eating disorder were also eligible for IOP intervention, however were not included in this study as none came through during the recruitment period that met the criteria.

MCCAED is a large, specialist child and adolescent ED service that sees young people below the age of 18 in London, UK, with a catchment area of over two million. See previous publications for a more detailed description of MCCAED outpatient and DP services [3, 54, 55]).

### Data collection

Demographic data were collected as part of routine clinical care. This included recorded notes, clinic letters, and referral information (including total duration of eating disorder and rate of weight loss before starting IOP). Treatment data, routinely collected by the service, were recorded from treatment sessions that took place in the IOP. Data recorded included age at the IOP assessment, gender, ethnicity, ICD-11 diagnosis, the IOP assessment and discharge date, weight (in kg), height (in m) and percentage of median body mass index (%mBMI) for the five weeks prior to referral to IOP, during IOP, and, at discharge from hospital and five weeks post-discharge from IOP, date of each anthropometric measurement, reason for discharge from the IOP, medications prescribed and duration of ED treatment prior to the IOP. Weights and heights were measured in the treatment centre by members of the treatment team competent in measuring and recording these parameters. All weights were measured at weekly intervals by clinicians using standard weighing procedures (i.e., weighing participants before meals, on the same calibrated scales, in light clothing, without shoes, with emptied pockets, and after toileting).

For cases that had at least one admission to a paediatric ward setting, additional data collected during the period of admission included duration of admission (in days), use of nasogastric (NG) tube feeding and duration of NG tube feeding (days).

### Self-report measures

A battery of validated psychometric questionnaires were routinely completed by both young people and their parents at the initial IOP assessment and at discharge (i.e. first and last contact, respectively) to assess any changes in symptom severity over the course of the intervention. Data derived from the following questionnaires was included for young people: Symptoms of anxiety and depression were measured using the 47-item Revised Children’s Anxiety and Depression Scale (RCADS) young person version [34]. The RCADS uses a 4-point scale (0 = “never”, 3 = “always”) to assess how often an individual has felt bothered by a range of symptoms, as measured by one depression subscale, five anxiety subscales (separation anxiety, generalised anxiety, panic disorder, social phobia, obsessive-compulsive) and a total summed internalizing scale. The RCADS has been used extensively and shown to have good psychometric properties.

Eating disorders symptoms were measured using the 12-item Eating Disorder Examination Questionnaire (EDE-QS) and Nine-Item ARFID Screen (NIAS) [21]. The EDE-QS uses a 4-point scale to assess severity of restricted eating disorder symptoms, measured on one total score. This has been used widely with young people with available adolescent norms. The NIAS uses a 5-point scale to assess the severity of three restrictive eating patterns (lack of interest in eating or food, sensory sensitivity and fear of aversive consequences) and total summed score, with higher scores indicating higher levels of avoidant/restrictive eating overall.

For parents, data were derived from the RCADS and the Depression, Anxiety and Stress Scales-21 (DASS-21). The DASS is a 42-item self-report instrument developed [46] to measure the three related negative emotional states of depression, anxiety and tension/stress. The DASS- 21 is a shortened form of the DASS-42 and includes 7 items from each of the 3 subscales. The 21-item version of the measure has fewer items, a cleaner factor structure, and smaller interfactor correlations than the DASS-42. The psychometric properties of the DASS and normative data for the DASS are described in detail in the DASS manual [46].

### Data analysis

All statistical analyses were conducted using SPSS (version 29.0; IBM Corp, 2022). Descriptive statistics were reported for key variables that summarise the presentation of young people receiving treatment with the IOP including clinical characteristics and details of the IOP treatment (i.e. length of treatment and number of contacts per week). Data from self-reported measures were analysed for young people, and their parents, that completed the program service.

A Shapiro–Wilk test was used to determine whether data were normally distributed. Paired sample t tests were used to compare mean differences between assessment and discharge data, including anthropometric measures and self-reported questionnaire measurements, for those normally distributed variables, whereas the Wilcoxon signed-rank test compared variables that did not follow normal distribution. Effect sizes for statistically significant results were reported using Cohen’s classification: d = 0.2 (small), d = 0.5 (medium) and d > 0.8 (large).

## Results

### IOP pathways

**Fig. 1:**
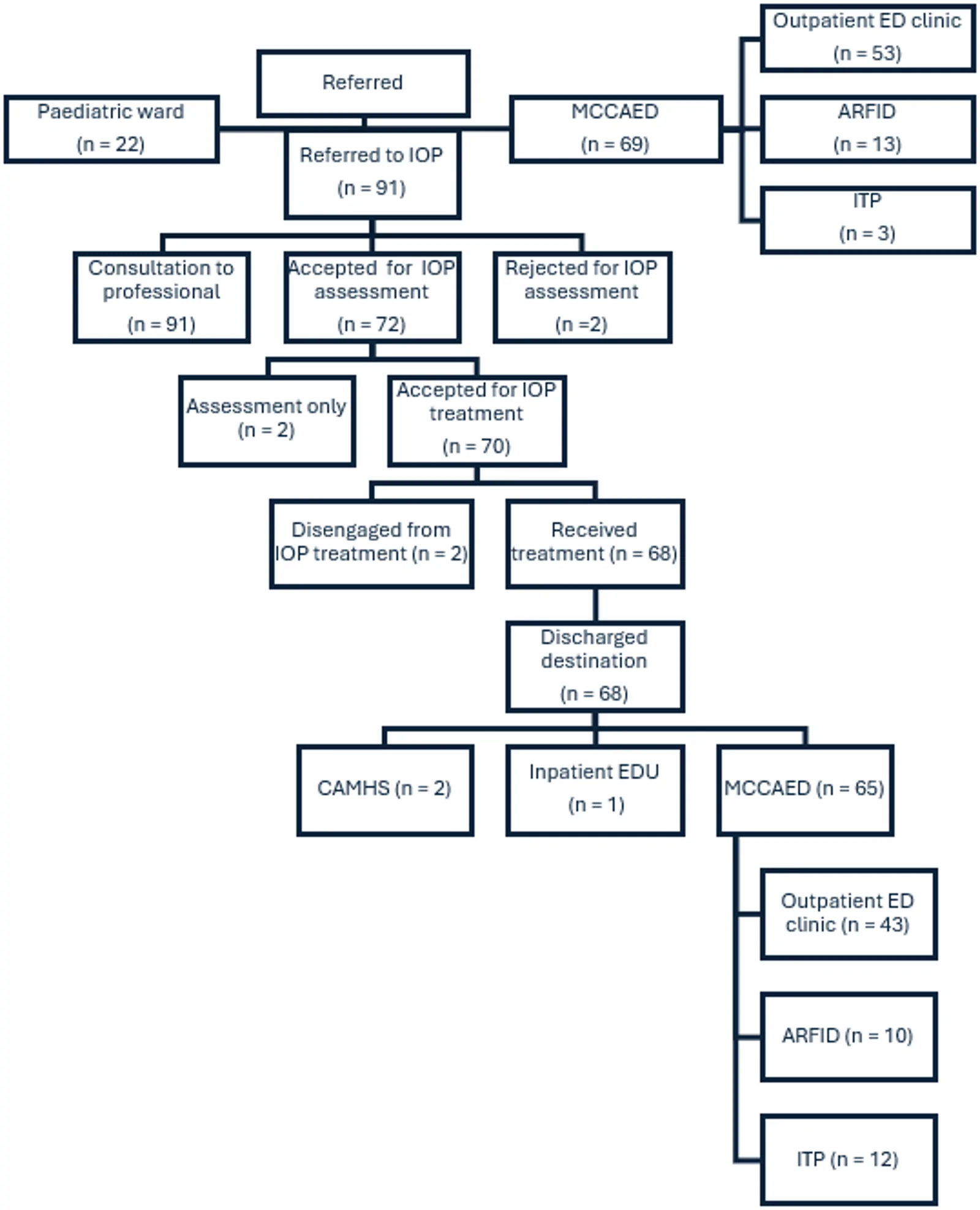
Flow diagram of the care pathways on referral and discharge from IOP

The total number of young people referred to the IOP consecutively between February 2024 and August 2025 comprised of 91 adolescents (mean age = 14.8, SD = 1.9 range = 11.8–17.5). (See Fig. 1 for IOP referral and treatment pathway). A majority (*n* = 69, 75.8%) of referrals came internally from other outpatient sub-teams within the wider MCCAED service, while the remainder (*n* = 22, 24.2%) of referrals to the IOP came from acute paediatric hospital sites in which the referred young people (previously unknown to MCCAED) had been admitted through the respective emergency department for medical stabilisation. Two (2.2%) referrals were not actioned due to not wanting to engage. In these two cases, parents/carers agreed with this decision and clinical risk could be adequately managed by the MCCAED team, without the need for higher levels of care.17 (18.7%) referrals received consultation to professionals only (in the absence of any direct contact with the young person or their carers). Professional consultations involved a member of the IOP taking part in one singular online meeting with staff external to the ED service seeking specialist guidance from the IOP team on general aspects of managing ED cases on an acute (non-ED specialist or psychiatric) ward.

The remaining 72 young people (79% of total referrals) were assessed by the IOP for eligibility to receive treatment. For two of these referrals, the IOP was no longer deemed a necessary intervention by the referring clinician. Of the 70 referrals accepted for treatment, two young people (2.9%) disengaged from the intervention within the first week (duration of IOP input < 6 days) and were discharged from the IOP back to the MCCAED outpatient ED clinic. Data for the remaining 68 referrals who were accepted and completed the IOP treatment was further analysed and described in this study. These 68 young people, after completion of the IOP treatment, were discharged to another part of the wider MCCAED service (total *n* = 65; eating disorder clinic *n* = 43, ARFID clinic *n* = 10, intensive day treatment programme team *n* = 12), to a SEDU (*n* = 1) or to a local child and adolescent mental health (CAMHS) team (*n* = 2).

### Sample characteristics

Descriptive statistics of the socio-demographic and clinical characteristics at the IOP assessment for all 68 young people who received treatment are presented in Table 2. Mean %mBMI at assessment was 80.22% and mean weight was 41.5 kg. Most cases self-identified as female (86.8%) and white British (52.9%). The majority had a diagnosis of AN (55.9%). The rest had a diagnosis of ARFID (14.9%), OSFED – Atypical AN (11.8%), BN (1.5%), or subthreshold disordered eating (16.2%). Those who did not have a primary ED diagnosis included cases presenting with disordered eating in the context of other mental health difficulties, requiring IOP treatment to reduce the need for an inpatient admission to treat their disordered eating symptoms. 75% of young people had ED treatment prior to the IOP assessment, with a mean duration of 148.3 days (SD = 181.4) of treatment.

44.1% and 16.2% of the young people had a diagnosis or were awaiting assessment for a diagnosis of autism spectrum disorder or attention deficit hyperactivity disorder (ADHD), respectively. 14.7% of the young people had a diagnosis of obsessive-compulsive disorder (OCD). 52.9% of young people were prescribed medication. See Table 2 for further details.

**Table 2:** Clinical characteristics and demographic details of young people treated by the IOP

		M (SD)
Weight	Kilograms	41.5 (8.4)
	%mBMI	80.1 (9.9)
Age	Years	14.8 (1.9)
		***N (%)***
Gender	Female	59 (86.8%)
	Male	8 (11.8%)
	Non-binary	1 (1.5%)
Ethnicity	White- British	36 (52.9%)
	White-Other	13 (19.1%)
	Black-British	7 (10.3%)
	Mixed-Ethnic Background	7 (10.3%)
	Asian British	3 (4.4%)
	Other Ethnic Group	2 (2.9%)
ED diagnosis*	Avoidant / Restrictive Food Intake Disorder (ARFID)	10 (14.7%)
	Anorexia Nervosa (AN)	38 (55.9%)
	Bulimia Nervosa (BN)	1 (1.5%)
	Other Specified Feeding or eating disorder (OSFED) – AAN	8 (11.8%)
	No primary eating disorder diagnosis	11 (16.2%)
Other diagnoses*	Autism Spectrum Disorder (ASD)	30 (44.1%)
	Attention Deficit Hyperactivity Disorder (ADHD)	11 (16.2%)
	Obsessive-Compulsive Disorder (OCD)	10 (14.7%)
Prior ED treatment	Yes	51 (75%)
	Duration,* days (SD)*	148.3 (181.4)
Medication	None	32 (47.1%)
	Olanzapine	27 (39.7%)
	Fluoxetine	6 (8.8%)
	Sertraline	1 (1.5%)
	Promethazine	2 (2.9%)

*All diagnosis as per ICD-11 classification

### Treatment course

**Fig. 2:**
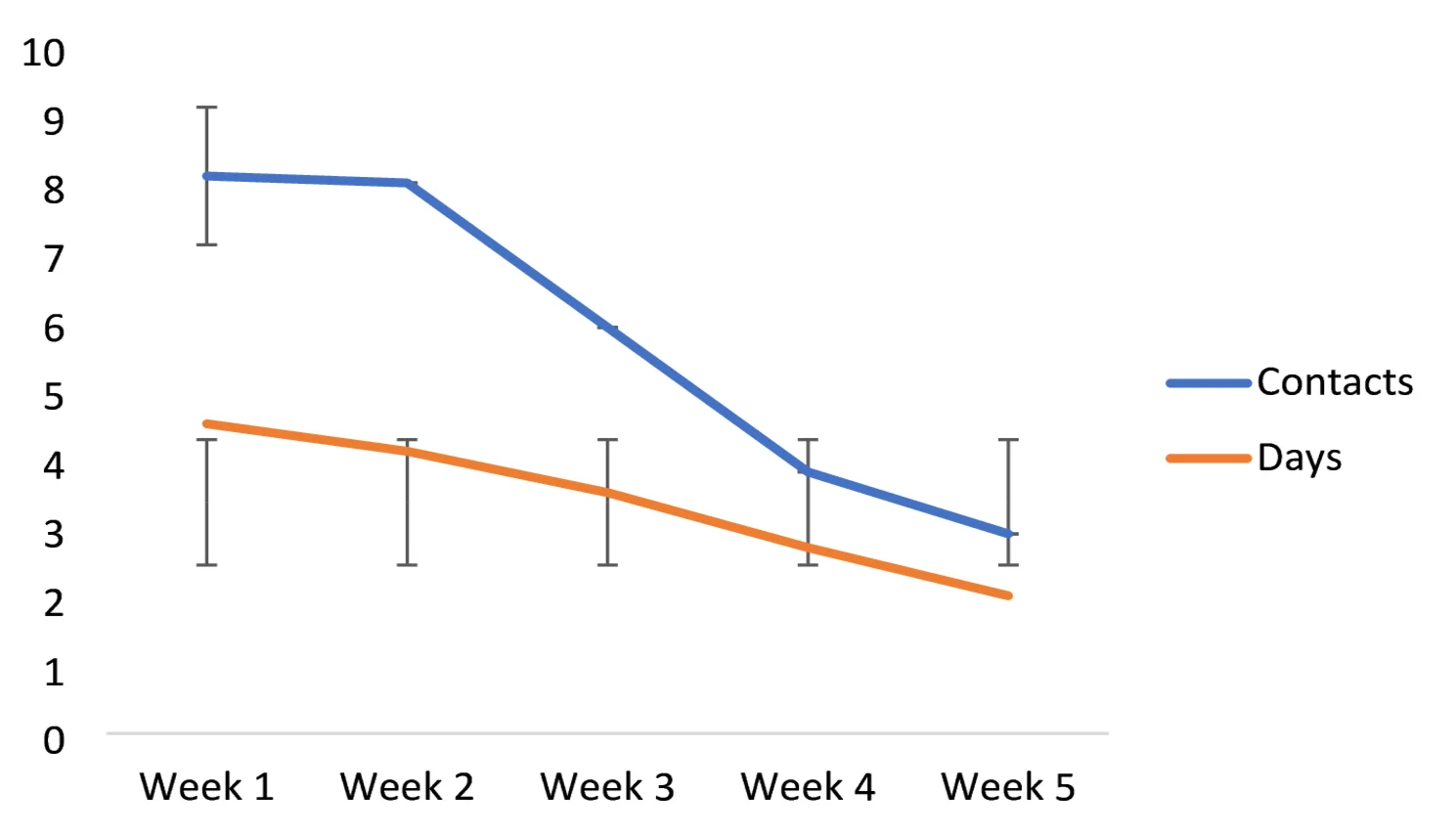
Frequency of sessions with the IOP over the 5-week period

Figure 2 contains details of the IOP treatment course, duration and intensity. The mean length of the IOP treatment was 23.7 (SD = 10.7) days over 3.4 weeks (SD = 1.5), with a mean of 24.12 (SD = 12.8) sessions. Three young people attended for one week only, 13 for 2 weeks, 18 for 3 weeks, 22 for 4 weeks, and 12 attended for additional days post week-4, spanning into an unplanned fifth week.

Half (50.0%) of the young people seen by the IOP required admission to an acute paediatric ward for medical stabilisation, with most (88.2%) admitted within the first week of the IOP. Of those admitted, 18 (52.9%) were already on a paediatric ward at the time of the IOP referral, 12 (35.3%) were referred simultaneously for paediatric admission and the IOP by the referring clinician, and only 4 (11.1%) were admitted after one week or more in the IOP. Three young people had multiple medical admissions (two admissions: *n* = 2; three admissions: *n* = 1), with the latter being the only young person requiring onward admission to a SEDU. The average hospital stay was 8.3 days (SD = 4.0). Among those admitted, 35.3% required nasogastric (NG) feeding for an average of 5.5 days (SD = 3.7).

### Treatment outcomes

**Fig. 3:**
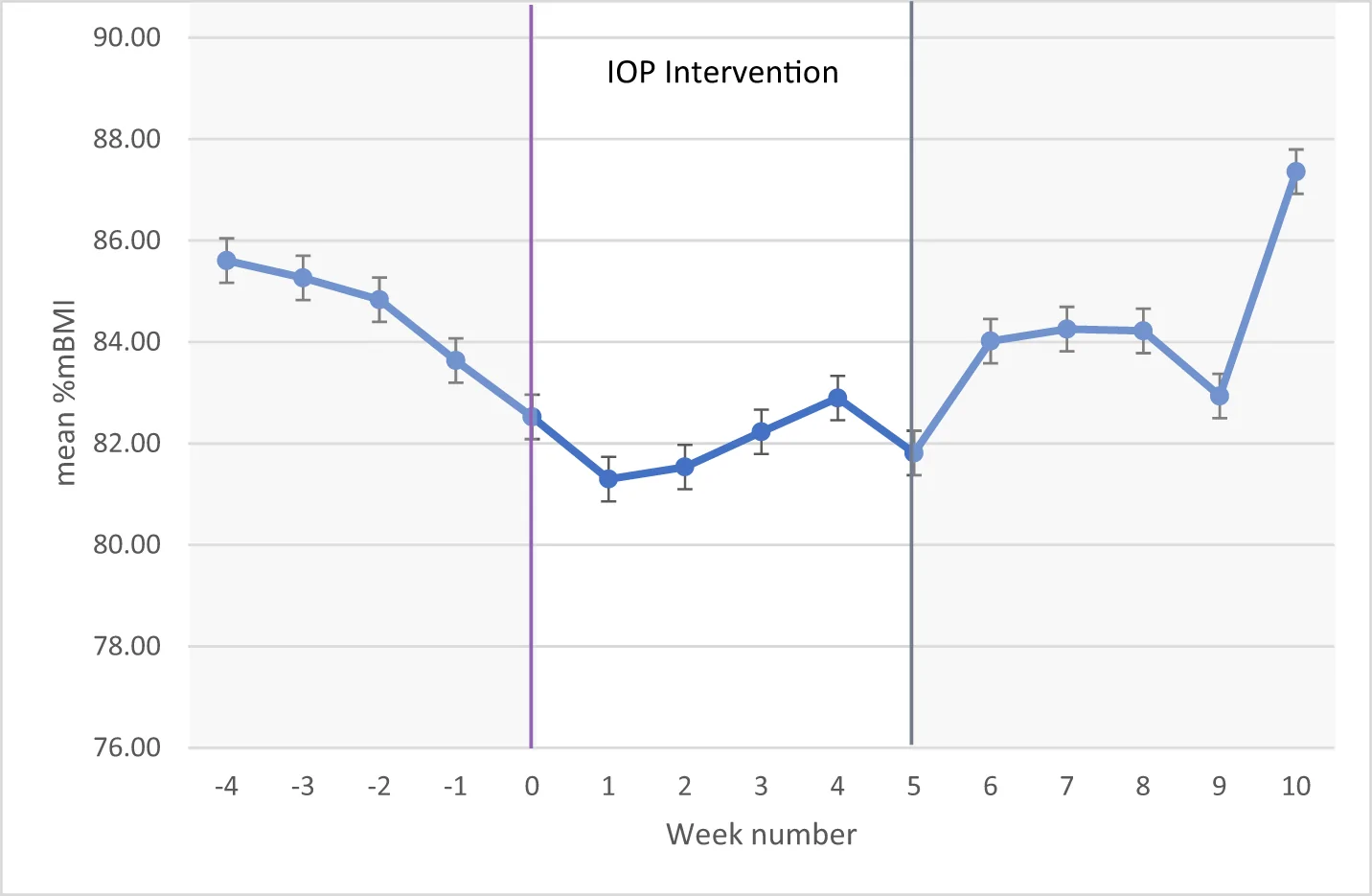
Trajectory of mean %mBMI for young people five weeks pre, during and after the IOP intervention. Pre = -4 to 1, during = 1 to 5, after = 5 to 10. *Standard IOP intervention was 4-weeks, a minority of patients required additional days that stretched into a fifth week which is represented in this table

Figure 3; Table 3 visualise the trajectory of weight change for young people over the five weeks preceding the IOP was steadily decreasing at a mean rate of -0.6%mBMI (SD = 0.8) per week. The decrease in weight trajectory ceased at week one of IOP and a significant increase in rate of weight change was identified over the five weeks during the IOP when compared with the five weeks pre the IOP (-0.6% vs. + 0.8%, *p* < 0.001). Post the IOP, the average trajectory of weight change continued to increase and no significant difference in rate of weight change was identified between the period during the IOP and the five weeks post (+ 0.8 vs. + 0.6, *p* = 0.494). See Fig. 3; Table 3 for further information.

**Table 3:** Rate of weight and %mBMI change for five weeks pre, during and post IOP intervention

Rate of change	Pre	During	*p*	d	During	Post	*p*	d
	M (SD)				M (SD)			
kg per week	-0.2 (0.3)	+ 0.5 (0.8)	< 0.001	-0.7	+ 0.5 (0.8)	+ 0.3 (0.6)	0.074	0.3
% per week	-0.6 (0.8)	+ 0.8 (1.2)	< 0.001	-0.7	+ 0.8 (1.1)	+ 0.6 (0.8)	0.498	0.2

In terms of overall change over the course of the IOP, there was a significant increase in weight over the course of IOP for the total sample (mean change = + 4.5%mBMI, SD = 6.8, d = -0.7). Sub-group analysis revealed that the largest changes were for the group with a diagnosis of AN (mean change = + 5.9%mBMI, SD = 8.2 d = 0.7). Significant weight gain was also observed for young people with ARFID (mean change = + 3.6%mBMI, SD = 4.2, d = 0.9) and no ED diagnosis (mean change = + 3.9%mBMI, SD = 4.9, d = 0.8). Weight did not significantly change for young people with OSFED-AAN across IOP treatment (mean change = + 1.2%mBMI, SD = 3.4, d = 0.4). See Table 4 for further details.

**Table 4:** Anthropometric outcomes of young people after completing IOP treatment

Diagnosis	Assessment	Discharge	Change	*P*	d
	Mean %mBMI (SD)				
AN (*n* = 37)	78.8 (9.16)	84.8 (9.02)	5.9 (8.2)	< 0.001	-0.7
ARFID (*n* = 10)	72.0 (7.89)	75.6 (6.64)	3.6 (4.2)	0.02	-0.9
OSFED-AAN (*n* = 8)	90.8 (6.21)	91.9 (5.45)	1.2 (3.4)	0.34	-0.4
No ED *(n* = 10)	82.6 (8.49)	85.9 (6.93)	3.9 (4.9)	0.03	-0.8
Total Sample *(n* = 65)	80.2 (9.9)	84.6 (9.1)	4.5 (6.8)	< 0.001	-0.7

*Excluded BN case (*n* = 1). Also excluded AN case (*n* = 1) and no primary ED (*n* = 1) due to missing data

No significant changes between pre and post IOP self-reported measures (totals and subscales), including the EDE-QS, RCADS and NIAS, were observed (see Table 5). Parents displayed a significant increase in anxiety post IOP intervention. No other significant changes in self-reported measures were observed (see Table 6).

**Table 5:** Paired differences in assessment and discharge self-reported measures of young people completing IOP treatment

	Missing	Assessment	Discharge	*p*	d
	*N* (%)	M (SD)			
EDE-Q global	23 (66.1)	15.7 (9.0)	14.7 (8.1)	0.447	0.2
NIAS	13 (80.9)	19.8 (10.1)	18.8 (9.5)	0.622	0.1
RCADS					
GAD	22 (67.6)	7.6 (4.1)	7.6 (3.8)	0.932	-0.1
Phobia	22 (67.6)	15.6 (5.1)	14.5 (6.6)	0.280	0.2
Depression	22 (67.6)	15.7 (6.0)	15.3 (6.4)	0.711	0.1
OCD	22 (67.6)	6.1 (4.6)	5.9 (4.2)	0.773	0.1
Separation anxiety	22 (67.6)	6.7 (3.8)	7.4 (4.7)	0.367	-0.2
Panic disorder	22 (67.6)	7.7 (5.2)	7.9 (6.5)	0.843	-0.1
Total anxiety	22 (67.6)	43.6 (18.0)	43.3 (21.3)	0.923	0.1
Total internalizing	22 (67.6)	59.40 (22.4)	58.6 (26.0)	0.859	0.1

*Excluded BN case *(n* = 1). Also excluded AN case (*n* = 1) and no primary ED case (*n* = 1) due to missing data

**Table 6:** Paired differences in assessment and discharge self-reported measures of parents of YP completing IOP treatment

	Missing *N* (%)	Assessment M (SD)	Discharge M (SD)	*p*	d
DASS-21					
Depression	47 (69)	17.4	18.0	0.966	0.1
Anxiety	50 (74)	16.4	31.7	0.010	-0.7
Stress	46 (67)	21.4	22.2	0.713	0.1
RCADS					
GAD	45 (66)	7.8	7.6	0.837	0.1
Phobia	45 (66)	14.2	14.3	0.921	-0.1
Depression	45 (66)	14.6	12.7	0.095	0.4
OCD	45 (66)	5.5	4.4	0.250	0.3
Separation anxiety	45 (66)	5.9	6.0	0.905	-0.1
Panic disorder	45 (66)	5.5	4.9	0.568	0.1
Total anxiety	45 (66)	38.9	37.3	0.609	0.1
Total internalizing	45 (66)	53.6	50.0	0.401	0.2

Upon completion of the IOP treatment, most young people (76.5%) continued eating disorder treatment as outpatients in MCCAED (ED clinic and ARFID clinic) (See Fig. 1). Twelve young people were referred to the intensive day treatment programme (17.6%) and one young person to a SEDU (1.5%). Two young people (2.9%) were discharged to Child and Adolescent Mental Health Services (CAMHS) for treatment of other, primary mental health difficulties.

## Discussion

This study describes the pathway and treatment course of a pilot, UK based, IOP intervention for children and adolescents with EDs. Our study aimed to identify the impact of this brief intervention on weight trajectory and ED symptoms for young people who completed the IOP treatment. Findings were consistent with other literature describing potential benefits of short-term, IOPs in supporting weight gain and therefore reducing the need for inpatient treatment [2, 52]. The lack of significant change in ED symptoms across the IOP intervention is, perhaps, unsurprising given the very brief nature of the IOP (mean duration = 24 days). The aim of the IOP is not to reach recovery, rather to support young people in crisis, to avoid inpatient SEDU admissions and to support outpatient treatment. This fits with principles of least restrictive intervention and align with how other UK models conceptualise intensive intervention as one component of a broader pathway [9]. It also fits with the broader literature that intensive, family-inclusive support that feels individualised and flexible can promote (re)engagement with the therapeutic process in outpatient treatments and support young people to make effective recovery-oriented change [6–8, 26]. From the current study it seems a brief IOP can function to offer quick, wrap-around support to young people and families to ensure they are able to remain in least-restrictive outpatient treatment. Additional markers of change that were not measured quantitatively nor reported here, included engagement in treatment and improved therapeutic alliance (please see our previous publications for further details on qualitative outcomes of this IOP) [16, 17]. The data also suggest that IOPs can act as a step in between outpatient treatment and more intensive, resource heavy, day programme treatment.

Most referrals to the IOP were internal, from the MCCAED service, suggesting that IOP primarily functioned as a step-up intervention for young people already known to the ED service who were needing more intensive support. A smaller proportion were referred from acute hospitals following emergency admission. Paediatric admissions which include specialist support, such as ED psychoeducation, parental meal coaching and behavioural contracting, are shown to improve parental self-efficacy and weight trajectory [50]. Collaborative working between professionals in both specialist ED and non-specialist acute paediatric services similarly aids improved outcomes in this group - reducing length of hospital stay and stopping onwards journey to SEDU by facilitating safe discharge to community ED services [56]. Our findings mirror those of current literature, indicating that the IOP served an important role in providing intreach ED care to paediatric wards, fafacilitating safe discharge back to community treatment settings.

The cohort observed in this study reflected patterns commonly seen in adolescent ED populations, with most participants identifying as female and White British, and with AN as the predominant diagnosis [40, 47]. Importantly, a minority did not have a primary ED diagnosis but presented with disordered eating in the context of other psychiatric difficulties, requiring acute hospital treatment. This underlines the clinical heterogeneity of referrals and suggests that IOP models may be suitable for young people beyond narrowly defined ED diagnostic categories [19].

Neurodevelopmental comorbidity in the current study was striking, with 44.1% of participants having or awaiting an autism spectrum disorder (ASD) diagnosis and 16.2% attention deficit hyperactivity disorder (ADHD). A further 14.7% had obsessive-compulsive disorder (OCD). These findings are consistent with growing evidence of high neurodevelopmental and psychiatric overlap in ED populations, particularly in restrictive presentations [58]. For services, this highlights the need for interventions that are adaptable to the bio-psychosocial profiles of neurodivergent adolescents, including difficulties with flexibility, sensory sensitivities, and impulsivity [44]. Increasingly, intervention to support people with neurodivergent people with EDS are emerging and showing promise [14, 23, 45]. Tailoring IOP programs to these needs may improve engagement and outcomes. Additionally, transdiagnostic interventsions may be an avenue to explore given early promise of these interventions for this population [12].

Clinically, significant weight gain (directly following a period of steady decline) was observed across the sample, with mean %mBMI increasing by 4.5% during treatment, at an average rate of 1.5% per week. This trajectory of weight continued post discharge from the IOP, without any significant change in rate between the period during the IOP and the five weeks post (see Fig. 3; Table 4 for further information).

Gains were most pronounced for young people with AN and ARFID, while those with atypical AN showed non-significant weight change, reflecting differences in treatment goals and physical health. Early weight gain, facilitated by swift nutritional rehabilitation, is the cornerstone of the initial stages of recovery in the treatment of restrictive EDs, resulting in improved clinical outcomes and shorter treatment duration [37] with greater weight gain during initial treatment (i.e., the first month) predicting full remission after one year [47]. Results pertaining to weight trajectory from this study are comparable to weight outcomes reported internationally for both partial hospitalisation and DPs [54, 57], supporting the utility of brief IOP as an effective intervention for supporting weight gain.

In contrast, minimal changes were observed in self-reported psychometric measures, with parental anxiety being the sole measure to significantly change (increase) over the course of the IOP intervention. Following discharge from the IOP, parental anxiety may increase as responsibility for supporting the YPs recovery shifts more fully to the home environment. Additionally, as the eating disorder behaviours are challenged during IOP treatment, distress can temporarily increase whilst the young person increases their dietary intake. Parental anxiety may therefore become elevated in response to this heightened distress. Longer follow up data is needed to better understand this increase in parental anxiety.

As aforementioned, the lack of change in other quantitiave measures is unsurprising given the short treatment duration likely providing insufficient time for psychological change to emerge and weight restoration preceding symptomatic improvements in underweight adolescents with ED [40]. Longer follow-up is needed to clarify whether psychological benefits emerge after discharge, particularly given IOP’s placing in the continuum of the ED care pathway rather than a stand-alone treatment [27].

### Strengths and limitations

This study provides one of the first systematic descriptions of adolescents receiving brief IOP for EDs in a UK specialist service. Strengths include prospective data collection, focus on both clinical and service-level outcomes, and inclusion of a diagnostically heterogeneous sample reflective of real-world practice.

A number of limitations also exist. Firstly, the study employed an uncontrolled design which limits the strength of results. Secondly, the inclusion of YP with diverse diagnoses that completed varying duration and intensitiy of IOP may limit interpretation of results. Thirdly, no longer term follow up from the respective IOP exists resulting in follow up data only extending to five-weeks post intervention. Additionally, the sample’s demographic homogeneity (predominantly female, White British) limits generalisability and lastly, very few participants presented with binge-purge symptoms. There is a general lack of evidence for effective treatments for this group [18]. Future research would benefit from better understanding the needs and most effective interventions for this group.

### Conclusion

IOPs fit the emerging UK service landscape, where intensive community and step-down models are positioned to bridge gaps between inpatient and community care [24]. Such services are expected not only to deliver direct treatment but also to support decision-making and care planning. The heterogeneity in referrals to the IOP evidenced the requirement for dynamic IOP support, tailoring the intervention to the clinical need. Professional consultation, outreach intervention on acute paediatric wards and intensified outpatient treatment were all key elements of the IOP that supported improved weight gain and therefore a reduction in the need for SEDU admissions.

Findings suggest that brief IOP intervention can be an effective option for adolescents with EDs, particularly for young people needing to restore weight. The program appeared to fulfil two roles: providing an intensification of care for those struggling in standard outpatient settings and offering an alternative to SEDU admission for those admitted to the paediatric ward. High rates of neurodevelopmental and psychiatric comorbidity reinforce the importance of flexible, tailored approaches to treatment delivery. Future research should include controlled designs, evaluate long-term outcomes, and explore adaptations for neurodivergent populations and people with non-restrictive EDs.

## Supplementary Information

Below is the link to the electronic supplementary material.

Supplementary Material 1.

## Data Availability

The datasets generated and analysed during the current study are not publicly available due ongoing data analysis as part of a wider study, but are available from the corresponding author on reasonable request.

## Abbreviations

%mBMI: Percentage median body mass index
AIM: Additional intervention and management group
ANOVA: Analysis of variance
ARFID: Avoidant restrictive food intake disorder
BN: Bulimia nervosa
CBT: Cognitive behavioural therapy
CBT-E: Cognitive behaviour therapy for eating disorders
CAMHS: Child and adolescent mental health service
ChOCI: Children’s obsessional compulsive inventory
CI: Confidence interval
ED: Eating disorder
EDE-Q: Eating disorder examination questionnaire
EDNOS-R: Eating disorder not otherwise specified predominantly characterised by restriction
EDQLS: Eating disorder quality of life scale
FT-AN: Family therapy for anorexia nervosa
FT-BN: Family therapy for bulimia nervosa
ICD 11: International classification of diseases (11th edition)
IQR: Interquartile range
ITP: Intensive treatment programme
MCCAED: Maudsley centre for child and adolescent eating disorders
MFT-AN: Multi-family therapy for anorexia nervosa
MFT-BN: Multifamily therapy for bulimia nervosa
MFQ: Mood and feelings questionnaire
NHS: National health system
QoL: Quality of life
RCT: Randomized controlled trial
SCARED: Screen for child anxiety related emotional disorders
SD: Standard deviation
SIV: Self-induced vomiting
SPSS: Statistical package for social sciences
SSRI: Selective serotonin reuptake inhibitor
UK: United Kingdom
USA: United States of America
