# Supplementary material for: The intensive outreach programme for child and adolescent eating disorders: an uncontrolled case series of clinical characteristics and treatment outcomes
Source: J Eat Disord. 2026 Jun 29;14:192. doi: 10.1186/s40337-026-01664-0 (PMC13487937; doi:10.1186/s40337-026-01664-0)
Supplement: Supplementary file 1 [file 40337_2026_1664_MOESM1_ESM.docx]

**Supplementary Information**

**IOP typical weekly activities and structure:**

*Week 1:*

Initial assessment takes place between the MCCAED treating therapist and an IOP clinician, alongside the parents/carer and young person. This may take place in clinic, or on the ward if the young person has already been admitted to hospital. Within this assessment, the treatment modality (i.e., FT-AN, FT-BN, ARFID treatment or adapted version of these treatments), current level of risk, formulation and goals for IOP are discussed collaboratively to generate an initial IOP treatment plan (i.e., intensity and content of the treatment).

Within the first week of the IOP, families are typically reviewed daily and most often in clinic (or on the ward if admitted). For some families, remote appointments can be facilitated, if daily physical monitoring is not necessary. Typical activities that take place in week 1 include: meal support and parent coaching (up to 3 times per day, daily), physical review (at least twice per week, and up to daily where clinically indicated), parent only sessions (daily, involving review of previous days and planning for future days) and dietetic review (where meal planning is necessary). For young people admitted to the paediatric ward, tailored support for managing the refeeding phase may also be required prior to them being discharged back to the community (sometimes requiring short term oral and/or enteral nutrition supplementation).

*Week 2:*

A joint review takes place between the MCCAED treating therapist, an IOP clinician and the family. Goals from week 1 are reviewed and further goals and expectations are set for week 2. Within the second week of the IOP, families are typically reviewed 3-4 times/ week in clinic (or ward), remote appointments can be facilitated, if daily physical monitoring is not necessary.

Typical activities that take place in week 2 include: meal support and parent coaching (up to 3 times per day, 3-4 times per week), physical review (at least twice per week, and up to daily where clinically indicated), parent only sessions (3-4 times per week, involving review of previous days and planning for future days) and dietetic review (where meal planning is necessary). For young people still admitted to the paediatric ward, specific support may be required in week 2 to move away from oral/enteral nutritional supplementation on to exclusive food-based nutrition. Individual support for the young person may also be offered in week 2 (dependent on the young person’s formulation), typically involving work on motivation, valued and/or distress tolerance skills.

*Week 3:*

Another weekly joint review takes place between the MCCAED treating therapist, an IOP clinician and the family. Goals from week 2 are reviewed and further goals and expectations are set for week 3. Within the third week of the IOP, families are supported to step down from the IOP and continue to implement change at home. Typically, being reviewed 2-3 times/ week in clinic (or ward), remote appointments are often facilitated, if daily physical monitoring is not necessary.

Typical activities that take place in week 3 include: meal support and parent coaching (up to 3 times per day, up to twice per week), physical review (at least once per week, or up to daily where clinically indicated), parent only sessions (twice per week, involving review of previous days and planning for future days) and dietetic review (where meal planning is necessary). In a minority of cases, some young people may remain admitted to the paediatric ward, in these cases discharge planning intensifies to support their transition back to the community. Individual support for the young person may also be offered in week 3 (dependent on the young person’s formulation), continuing themes from week 2.

*Week 4:*

A final joint review takes place between the MCCAED treating therapist, an IOP clinician and the family. Progress over the course of the IOP, overall goals and strategies/techniques that have aided progress are discussed. Within the fourth week of the IOP, families are supported to step down from the IOP and continue to implement change at home. Typically, being reviewed 1-2 times/ week in clinic (or ward), remote appointments are often facilitated, and daily physical monitoring is not usually necessary.

Typical activities that take place in week 4 include: meal support and parent coaching (1-2 meals per day, usually 1/week), physical review (often once per week, and sometimes not necessary by the final week at all), parent only sessions (once per week, involving review of previous days and planning for future days) and dietetic review (not usually required in the final week). In exceptional cases, young people may remain admitted to the paediatric ward, in these cases discharge planning may involve planning transfer to a specialist eating disorder or mental health bed if discharge to the community is not appropriate. Individual support for the young person may also be offered in week 4 (dependent on the young person’s formulation), continuing themes from week 3.

Most families are successfully discharged from the IOP by the end of week 4. Decisions around discharge are collaborative, involving stepping down over previous weeks (so that ending do not feel abrupt) and planning next steps in treatment with the MCCAED treating clinician. For some families, an extension of the IOP may be needed and in these cases additional days/weeks typically involve activities similar to those in week 3 and 4, supporting a more gradual transition to standard outpatient care.
